# Estimands to quantify prolonged hospital stay associated with nosocomial infections

**DOI:** 10.1186/s12874-019-0752-6

**Published:** 2019-05-31

**Authors:** Martin Wolkewitz, Martin Schumacher, Gerta Rücker, Stephan Harbarth, Jan Beyersmann

**Affiliations:** Institute of Medical Biometry and Statistics, Faculty of Medicine and Medical Center - University of Freiburg, Stefan-Meier 26, Freiburg, Germany

**Keywords:** Multi-state model, Hospital-acquired infections, Sojourn time, Length of hospital stay

## Abstract

**Background:**

Length of stay evaluations are very common to determine the burden of nosocomial infections. However, there exist fundamentally different methods to quantify the prolonged length of stay associated with nosocomial infections. Previous methodological studies emphasized the need to account for the timing of infection in order to differentiate the length of stay before and after the infection.

**Methods:**

We derive four different approaches in a simple multi-state framework, display their mathematical relationships in a multiplicative as well as additive way and apply them to a real cohort study (n=756 German intensive-care unit patients of whom 124 patients acquired a nosocomial infection).

**Results:**

The first approach ignores the timing of infection and quantifies the difference of eventually infected and eventually uninfected; it is 12.31 days in the real data. The second approach compares the average sojourn time with infection with the average sojourn time of being hypothetically uninfected; it is 2.12 days. The third one compares the average length of stay of a population in a world with nosocomial infections with a population in a hypothetical world without nosocomial infections; it is 0.35 days. Finally, approach four compares the mean residual length of stay between currently infected and uninfected patients on a daily basis; the difference is 1.77 days per infected patient.

**Conclusions:**

The first approach should be avoided because it compares the eventually infected with the eventually uninfected, but has no prospective interpretation. The other approaches differ in their interpretation but are suitable because they explicitly distinguish between the pre- and post-time of the nosocomial infection.

## Introduction

Length of stay (LOS) is one of the most important outcomes in clinical epidemiology since it is directly linked to patients’ morbidity and economic costs [1]. It is easy to measure and often routinely collected in surveillance data bases. During the stay in hospital, patients are at risk to acquire nosocomial infections (NI) which belong to the major common adverse events in hospitals. Many observational reports have studied the impact of NI on length of stay by using different statistical methods. When evaluating the prolonged LOS of NI, the timing of NI plays an important role to distinguish between pre-infection time and consequence of NI. Several methodological papers showed the magnitude of the so-called time-dependent (aka immortal-time) bias which occurs if the timing of infection is not adequately addressed or rather ignored in the analysis [2, 3]. Multi-state models or time-dependent matching techniques account for the timing of NI to avoid the time-dependent bias [2–4]. However, there exist fundamentally different estimands to quantify this prolonged LOS associated with NI. In this article, we describe four different approaches and estimands in a simple multi-state framework [5], display their mathematical relationships in a multiplicative as well as additive way and apply them to a real cohort study.

## Methods

**Fig. 1:**
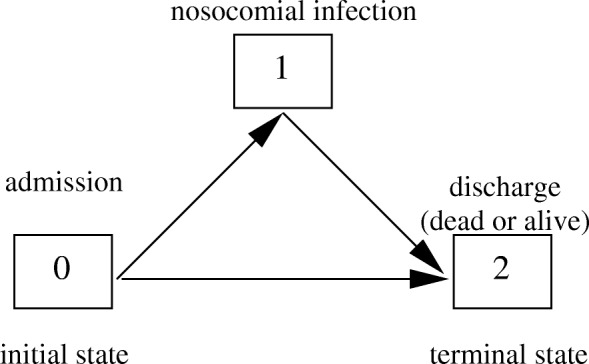
Multi-state model

We consider a time-homogeneous multi-state model (Fig. 1) with the three states 0=admission, 1=nosocomial infection, 2=discharge/death and assume constant hazard rates *λ*_01_,*λ*_02_ and *λ*_12_ between the corresponding states. The hazards *λ*_*ij*_ are interpreted as the daily risk of moving from state *i* to state *j*.

The infection hazard *λ*_01_, also denoted as the incidence density of NI, is estimated by dividing the number of NI events by the number of summed patient-days in state 0 [5]. Analogously, the event densities *λ*_02_ and *λ*_12_ are estimated by dividing the discharge/death events by the number of summed patient-days in state 0 and 1, respectively [5]. These estimates are formally obtained via maximum likelihood estimation [6]. Since the hazard rates are assumed to be time-constant, the time to leave state 0 follows an exponential distribution with the hazard rate *λ*_01_+*λ*_02_. Thus, the average sojourn time in state 0 is $\frac {1}{\lambda _{01}+\lambda _{02}}$. Analogously, the time to leave state 1 follows an exponential distribution with the hazard rate *λ*_12_ leading to an average sojourn time in state 1 of $\frac {1}{\lambda _{12}}$. The probability to acquire a NI is equal to $\frac {\lambda _{01}}{\lambda _{01}+\lambda _{02}}$.

We can write the average LOS in terms of the hazards from the multi-state model, which is the sum of the sojourn time in state 0 and the sojourn time in state 1 multiplied with the probability to acquire a NI:

$$\begin{array}{*{20}l} \overline{LOS}=\frac{1}{\lambda_{01}+\lambda_{02}} + \frac{\lambda_{01}}{\lambda_{01}+\lambda_{02}} \times \frac{1}{\lambda_{12}} \end{array} $$

If we assume the common case that NI reduce the discharge hazard, i.e. $\frac {\lambda _{12}}{\lambda _{02}}<1$, we can use some algebra to derive following relationship:

$$\begin{array}{*{20}l} \frac{1}{\lambda_{02}} < \overline{LOS} < \frac{1}{\lambda_{12}} \end{array} $$

where $\frac {1}{\lambda _{02}}$ is interpreted as the average LOS in a hypothetical world without NI. The first inequality is shown as $\frac {1}{\lambda _{0}2}<\overline {LOS} \Leftrightarrow \lambda _{12}(\lambda _{01}+\lambda _{02})<\lambda _{02}(\lambda _{12}+\lambda _{01}) \Leftrightarrow \lambda _{12}\lambda _{01}<\lambda _{02}\lambda _{01} \Leftrightarrow \lambda _{12}<\lambda _{02}$. The second inequality is shown as $\overline {LOS}=(\lambda _{12}+\lambda _{01})/((\lambda _{01}+\lambda _{02})\lambda _{12})<(\lambda _{02}+\lambda _{01})/((\lambda _{01}+\lambda _{02})\lambda _{12}=1/\lambda _{12}$

These inequalities mean that the mean LOS in a world without NI is smaller than the mean LOS in a real world and this is smaller than the mean sojourn time in state 1. Based on the multi-state model, four different approaches to quantify the LOS associated with NI can be derived.

### Restrospective stratification of eventually infected and uninfected

The most common approach (A1) is to compare the average overall LOS of eventually infected patients with the average overall LOS of eventually uninfected patients. It addresses the following medical question of interest (see Table 1): ’How many days do patients with NI stay, on average, eventually longer in hospital than patients who will never acquire a NI?’. In terms of the multi-state model, the average overall LOS of eventually infected patients is the sum of the average sojourn times of state 0 and state 1, $\frac {1}{\lambda _{01}+\lambda _{02}}+\frac {1}{\lambda _{12}}$. The average overall LOS of eventually uninfected patients is the sojourn time in state 0, $\frac {1}{\lambda _{01}+\lambda _{02}}$. Thus, approach *A*_1_ is mathematically expressed by

$$\begin{array}{*{20}l} A_{1}&=\left(\frac{1}{\lambda_{01}+\lambda_{02}}+\frac{1}{\lambda_{12}}\right)-\left(\frac{1}{\lambda_{01}+\lambda_{02}}\right)=\frac{1}{\lambda_{12}}\\ &=\text{LOS difference of eventually infected and} \\&\quad\quad\quad \text{eventually uninfected} \end{array} $$

**Table 1:** Medical question of interest

Approach	Medical question of interest / meaning of corresponding estimand
*A*_1_	- How many days do patients with NI stay, on average, eventually longer in hospital than patients who will never acquire a NI?
	- How many days do patients with NI stay, on average, after the NI?
*A*_2_	- How many hospital days are, on average, attributable to NI per patient?
	- How many hospital days, on average, would a patient have stayed shorter if he/she would not have acquired a NI?
*A*_3_	- How many hospital days are, on average, attributable to NI in a hospital population?
	- How many hospital days would the average length of stay be shorter if all NI in the population would be eliminated?
*A*_4_	- How many days does a patient with NI stay, on average, longer in hospital?
	- How many days, on average, is the expected prolonged stay for patients with NI?

which is the average sojourn time in state 1, given the patient has reached this state, i.e., has acquired a NI. In this approach, the classification of infected and uninfected is done retrospectively *at the end of hospital stay*.

This approach does not distinguish between pre- and post-infection LOS and thus does not allow a prospective associational (or even causal) interpretation. The limitation is that the LOS before NI which is included in the LOS of eventually infected patients can not be interpreted as LOS *attributed due to NI*. Instead, the LOS before NI (which is per-se not attributable to NI) should count as *uninfected* LOS. Therefore, the following approaches have been developed.

### Differentiating between pre- and post-infection length of stay

In contrast to the previous approach, the following approaches will differentiate between the pre-infection time and consequence of NI in terms of LOS.

The second approach *A*_2_, termed as attributable LOS [7, 8], compares the average sojourn time in state 1 $\left (\frac {1}{\lambda _{12}}\right)$ with the average sojourn time in state 0 in a hypothetical world without NI $\left (\frac {1}{\lambda _{02}}\right)$. The medical question of interest (see Table 1) is ’How many hospital days, on average, would a patient have stayed shorter if he/she would not have acquired a NI?’.

This is quantified by

$$\begin{array}{*{20}l} A_{2}&=\frac{1} {\lambda_{12}} - \frac{1}{\lambda_{02}} \\ &= \text{Attributable length of stay} \end{array} $$

In contrast to approach *A*_1_, the left part of the difference in approach $A_{2} \left (\frac {1} {\lambda _{12}}\right)$ considers only the post-infection LOS of infected patients. Moreover, the right part $\left (\frac {1}{\lambda _{02}}\right)$ considers a longer LOS than the one of approach *A*_1_ as $\frac {1}{\lambda _{02}}> \frac {1}{\lambda _{01}+\lambda _{02}}$. This corrects for the limitations of approach *A*_1_. However, the limitation of approach *A*_2_ is that $\frac {1}{\lambda _{02}}$ is not a real world mean time and is therefore a *hypothetical* quantity for LOS of uninfected patients.

In the third approach *A*_3_, we substract the average LOS in a hypothetical world from the one in a real world addressing the medical question ’How many hospital days would the average length of stay be shorter if all NI in the population would be eliminated?’. Algebraically, it is

$$\begin{array}{*{20}l} A_{3}&=\overline{LOS}-\frac{1}{\lambda_{02}}\\ &= \frac{\lambda_{01}}{\lambda_{01}+\lambda_{02}} \times \frac{1}{\lambda_{12}} \times \frac{(\lambda_{02}-\lambda_{12})} {\lambda_{02}}\\ &=\text{Population-attributable length of stay} \end{array} $$

This estimand is called the population-attributable LOS [8, 9], which is a *population measure* of extra LOS and compares the average LOS of a population in a world with NI with a population in a hypothetical world without NI.

In the fourth approach *A*_4_, we subtract the average length of stay from the sojourn time in state 1 which aims to answer the medical question ’How many days does a patient with NI stay, on average, longer in hospital?’ (see Table 1). It is

$$\begin{array}{*{20}l} A_{4}&= \left(\frac{\lambda_{02}} {\lambda_{12}}-1 \right) \times \frac{1}{\lambda_{01}+\lambda_{02}} \\ &=\text{Residual LOS of currently infected vs.}\\ &\quad\quad \text{currently uninfected } \end{array} $$

This estimand, also called the change of length of stay, is the established multi-state approach [10] and compares mean residual LOS between currently infected and uninfected patients using landmarking on each day in the hospital, it is a difference *per infected patient*.

### Basic properties related to the hazard ratio $\frac {\lambda _{12}}{\lambda _{02}}$

In this section we consider basic relationships to the hazard ratio $\frac {\lambda _{12}}{\lambda _{02}}$. The hazard ratio $\frac {\lambda _{12}}{\lambda _{02}}$ is often calculated and it describes in a multiplicative way if and how NI prolongs LOS. A hazard ratio of 1 means that the daily chance to be discharged does not change if the patient acquires a NI meaning that NI does not prolong the LOS. It is more often the case that the hazard ratio is smaller than 1 indicating a prolonged LOS associated with NI. It is rarely the case that the hazard ratio is greater than 1 which would mean a shortened LOS associated with NI. It is obvious that approach *A*_1_ is always larger than 0 (*A*_1_>0) as *λ*_12_>0. Since it further does not depend on *λ*_02_, *A*_1_ always means that NI patients stay eventually longer than patients who never acquired NI, even if $\frac {\lambda _{12}}{\lambda _{02}}=1$ or $\frac {\lambda _{12}}{\lambda _{02}}>1$ which is not a required property. For the other approaches we have: $\frac {\lambda _{12}}{\lambda _{02}}=1 \Leftrightarrow A_{2}=0 \Leftrightarrow A_{3}=0 \Leftrightarrow A_{4}=0$. It is also easily shown that $\frac {\lambda _{12}}{\lambda _{02}}<1 \Leftrightarrow A_{2}>0 \Leftrightarrow A_{3}>0 \Leftrightarrow A_{4}>0$ and $\frac {\lambda _{12}}{\lambda _{02}}>1 \Leftrightarrow A_{2}<0 \Leftrightarrow A_{3}<0 \Leftrightarrow A_{4}<0$. Thus, approaches *A*_2_, *A*_3_ and *A*_4_ have the required mathematically equivalent properties regarding the direction of the hazard ratio $\frac {\lambda _{12}}{\lambda _{02}}$ whereas approach *A*_1_ does not.

In Table 2, the properties of all approaches are displayed, summarized and contrasted to each other.

**Table 2:** Approaches and their properties

Approach	Properties / pros and cons
*A*_1_	- is a real quantity
	- undesired properties related to hazard ratio $\frac {\lambda _{12}}{\lambda _{02}}$
	- yields positive values even if NI patients are discharged faster, i.e., $\frac {\lambda _{12}}{\lambda _{02}}>1$
	- does not distinguish between pre- and post-infection time
	- does not allow causal interpretation about attributable length of stay associated with NI
	- not appropriate to quantify the burden of NI
*A*_2_	- is a hypothetical quantity
	- desired properties related to hazard ratio $\frac {\lambda _{12}}{\lambda _{02}}$
	- considers only the post-infection time for NI patients
	- contributes pre-infection time to patients without NI
	- allows a causal interpretation about attributable length of stay associated with NI
	- appropriate to quantify the burden of NI at patient-level
*A*_3_	- is a hypothetical quantity
	- desired properties related to hazard ratio $\frac {\lambda _{12}}{\lambda _{02}}$
	- considers only the post-infection time for NI patients
	- contributes pre-infection time to patients without NI
	- allows a causal interpretation about attributable length of stay associated with NI
	- appropriate to quantify the burden of NI at population-level
*A*_4_	- is a real quantity
	- desired properties related to hazard ratio $\frac {\lambda _{12}}{\lambda _{02}}$
	- distinguishes between pre- and post-infection time
	- appropriate to quantify the burden of NI at patient-level

### Additive and multiplicative comparisons of approaches

Before we compare the approaches in a additive and multiplicative way, we note that approaches *A*_1_ and *A*_2_ do not depend on infection hazard *λ*_01_ whereas approaches *A*_3_ and *A*_4_ do. Further, approaches *A*_1_, *A*_2_ and *A*_4_ are at the patient-individual level and therefore directly comparable whereas *A*_3_ is at the population-level. All approaches are displayed in the Table 3. We further note that there is also following relationship: *A*_3_+*A*_4_=*A*_2_.

**Table 3:** Overview and relationships of approaches to quantify prolonged hospital stay associated with nosocomial infections

Approach	real data example (SIR-3 study)
	$\hat {\lambda }_{01}=124/6442 \approx 0.0192$
	$\hat {\lambda }_{02}=(756-124)/6442 \approx 0.0981$
	$\hat {\lambda }_{12}=124/1527 \approx 0.0812$
$A_{1}=\frac {1}{\lambda _{12}}$	12.31 days
$A_{2}=\frac {1} {\lambda _{12}} - \frac {1}{\lambda _{02}}$	2.12 days
$A_{3}=\frac {\lambda _{01}}{\lambda _{01}+\lambda _{02}} \times \frac {1}{\lambda _{12}} \times \frac {(\lambda _{02}-\lambda _{12})} {\lambda _{02}}$	0.35 days
$A_{4}=(\frac {\lambda _{02}} {\lambda _{12}}-1) \times \frac {1}{\lambda _{01}+\lambda _{02}}$	1.77 days
Additive relationships between approaches (differences)	
$A_{1}-A_{4}=\frac {1}{\lambda _{12}} \times \frac {\lambda _{01}+\lambda _{12}}{\lambda _{01}+\lambda _{02}}$	10.54 days
$A_{1}-A_{3}=\frac {1}{\lambda _{02}} \times \frac {\lambda _{02}^{2}+\lambda _{12}\lambda _{01}} {\lambda _{12}\lambda _{02}+\lambda _{12}\lambda _{01}}$	11.97 days
$A_{1} - A_{2}=\frac {1}{\lambda _{02}}$	10.19 days
$A_{4}-A_{3}=\frac {\lambda _{02}-\lambda _{01}}{\lambda _{02}} \times \frac {\lambda _{02}-\lambda _{12}}{\lambda _{12}(\lambda _{01}+\lambda _{02})} $	1.43 days
$A_{2}-A_{3}=A_{4}=\frac {\lambda _{02}-\lambda _{12}}{\lambda _{12}(\lambda _{01}+\lambda _{02})}$	1.77 days
$A_{2} - A_{4}=A_{3}=\frac {\lambda _{01}}{\lambda _{02}}\times \frac {\lambda _{02}-\lambda _{12}}{\lambda _{12}(\lambda _{01}+\lambda _{02})} $	0.35 days
Following relationship holds: *A*_3_+*A*_4_=*A*_2_	
Multiplicative relationships between approaches (ratios)	
$\frac {A_{1}}{A_{4}}=\frac {\lambda _{01}+\lambda _{02}}{\lambda _{02}-\lambda _{12}} \ge 1$	6.94
$\frac {A_{1}}{A_{3}}=\frac {\lambda _{02}(\lambda _{01}+\lambda _{02})} {\lambda _{01}(\lambda _{02}-\lambda _{12})} \ge 1$	35.4
$\frac {A_{1}}{A_{2}}=\frac {\lambda _{02}}{\lambda _{02}-\lambda _{12}} \ge 1$	5.80
$\frac {A_{3}}{A_{4}}=\frac {\lambda _{01}}{\lambda _{02}}= \text {odds(NI)} $	0.196
$\frac {A_{3}}{A_{2}}=\frac {\lambda _{01}}{\lambda _{01}+\lambda _{02}}=\text {risk(NI)} \le 1$	0.164
$\frac {A_{2}}{A_{4}}=\frac {\lambda _{01}+\lambda _{02}}{\lambda _{02}}=\frac {\text {odds(NI)}}{\text {risk(NI)}} \ge 1$	1.20
Following relationship holds if *λ*_01_≤*λ*_02_:*A*_3_≤*A*_4_≤*A*_2_≤*A*_1_	

#### Comparing approaches *A*_1_ and *A*_2_

The additive relationship between approaches *A*_1_ and *A*_2_ is just the average length of stay of a population in a hypothetical world without NI $\left (\frac {1}{\lambda _{02}}\right)$. The multiplicative relationship is $\frac {A_{1}}{A_{2}}=\frac {\lambda _{02}}{\lambda _{02}-\lambda _{12}}$.

#### Comparing approaches *A*_1_ and *A*_3_

As before, the relationship between approaches *A*_1_ and *A*_3_ is best described and communicable in an additive way. The difference between *A*_1_ and *A*_3_ is $A_{1}-A_{3}=\frac {1}{\lambda _{02}} \times \frac {\lambda _{02}^{2}+\lambda _{12}\lambda _{01}} {\lambda _{12}\lambda _{02}+\lambda _{12}\lambda _{01}}$ (Table 3). It is mainly the average length of stay of a population in a hypothetical world without NI $\left (\frac {1}{\lambda _{02}}\right)$; multiplied with the factor $\frac {\lambda _{02}^{2}+\lambda _{12}\lambda _{01}} {\lambda _{12}\lambda _{02}+\lambda _{12}\lambda _{01}}$ which is often larger than 1 as *λ*_02_ is often larger than *λ*_12_. In contrast, the multiplicative relationship is $\frac {A_{1}}{A_{3}}=\frac {\lambda _{02}(\lambda _{01}+\lambda _{02})} {\lambda _{01}(\lambda _{02}-\lambda _{12})}$.

#### Comparing approaches *A*_1_ and *A*_4_

The relationship between approaches *A*_1_ and *A*_4_ is best described in an additive way. The difference is $A_{1}-A_{4}=\frac {1}{\lambda _{12}} \times \frac {\lambda _{01}+\lambda _{12}}{\lambda _{01}+\lambda _{02}}=\overline {LOS}$ (Table 3). Thus, it mainly depends on the average sojourn time of state 1 which is $\frac {1}{\lambda _{12}}$; the factor $\frac {\lambda _{01}+\lambda _{12}}{\lambda _{01}+\lambda _{02}}$ is usually lower than 1 as *λ*_02_ is often larger than *λ*_12_. The multiplicative relationship is $\frac {A_{1}}{A_{4}}=\frac {\lambda _{01}+\lambda _{02}}{\lambda _{02}-\lambda _{12}}$.

#### Comparing approaches *A*_2_ and *A*_3_

Approaches *A*_3_ and *A*_2_ are best compared in a multiplicative way: $\frac {A_{3}}{A_{2}}=\frac {\lambda _{01}}{\lambda _{01}+\lambda _{02}}=\text {risk(NI)}$. The additive comparison is $A_{2}-A_{3}=A_{4}=\frac {\lambda _{02}-\lambda _{12}}{\lambda _{12}(\lambda _{01}+\lambda _{02})}$.

#### Comparing approaches *A*_2_ and *A*_4_

Approach *A*_2_ is linked to approach *A*_4_ with $\frac {A_{2}}{A_{4}}=\frac {\lambda _{01}+\lambda _{02}}{\lambda _{02}}=\frac {\text {odds(NI)}}{\text {risk(NI)}}$. Based on this formula, it follows that both approaches give similar results if the nosocomial infection is rare (risk is smaller than 10%). The additive comparison is $A_{2} - A_{4}=A_{3}=\frac {\lambda _{01}}{\lambda _{02}}\times \frac {\lambda _{02}-\lambda _{12}}{\lambda _{12}(\lambda _{01}+\lambda _{02})}$.

#### Comparing approaches *A*_3_ and *A*_4_

In contrast to the previous comparisons to approach *A*_1_, approaches *A*_3_ and *A*_4_ are best compared in a multiplicative way. There is the following simple relationship: $\frac {A_{3}}{A_{4}}=\frac {\lambda _{01}}{\lambda _{02}}= \text {odds(NI)}$. Thus, the factor odds(NI) links the population-level approach *A*_3_ to the individual-level approach *A*_4_. The additive relationship is rather complex: $A_{4}-A_{3}=\frac {\lambda _{02}-\lambda _{01}}{\lambda _{02}} \times \frac {\lambda _{02}-\lambda _{12}}{\lambda _{12}(\lambda _{01}+\lambda _{02})}$.

### Real data example

We use publicly available data from the R-package etm [11]. This is an observational prospectively collected cohort study including 756 intensive care patients from Germany of whom 124 patients acquired a nosocomial pneumonia (NI) during their stay in the intensive care unit (ICU). The data used here is a random sample from a larger cohort which is described in detail elsewhere [12].

## Results

The cohort study followed 756 patients during their stay at the intensive-care unit. Of these patients, 124 patients acquired a NI during their stay in the ICU. The summed patient-days without NI was 6442 and with NI 1527. Thus, the constant hazards can be estimated as follows: $\hat {\lambda }_{01}=124/6442=0.0192$, $\hat {\lambda }_{02}=632/6442=0.0981$, and $\hat {\lambda }_{12}=124/1527=0.0812$. The average length of stay of this cohort is $\overline {LOS}=10.54$ days; the sojourn time in state 0 is $\frac {1}{\hat {\lambda }_{01}+\hat {\lambda }_{02}}=8.52$ days and $\frac {1}{\hat {\lambda }_{12}}=12.31$ days in state 1, respectively. The results of the different approaches are diverse, however, with different interpretation. As expected, *A*_1_ provides a exaggerated value of 12.31 days which just means that patients with NI stay eventually 12.31 days longer at ICU then eventually uninfected patients. In contrast, *A*_4_ yields that a NI prolongs the LOS by mean 1.77 ICU days per infected patient. The population-attributable LOS (*A*_3_) is 0.35 days meaning that there are on average 0.35 additional ICU days attributable to NI at the population level. The attributable LOS (*A*_2_) is 2.12 ICU days, interpreted as the average LOS which is attributable to NI for an infected patient.

## Discussion

A multi-state model was used to mathematically derive four fundamentally different approaches to quantify the prolonged length of stay associated with nosocomial infections or other adverse events [13]. The relationships were displayed in an additive as well as a multiplicative way.

As in previous articles [2, 4], we encourage researchers to not retrospectively stratify by infection status and, consequently, to avoid the use of approach *A*_1_ because it does not differentiate between pre- and post-infection time and thus does not allow a causal interpretation.

The other approaches are suitable because they implicitly distinguish between the pre-infection time (which might be a risk factor) and post-infection time (which might be a consequence) of nosocomial infections. The main difference is the interpretation and we showed mathematical formulas how they are linked to each other. Thus, this knowledge can be used to better understand apparent discrepancies in the literature and transfer published values from one approach to the other.

The question whether nosocomial infections prolong hospital stay is - from the methodological point of view - related to ’life years lost among patients with a given disease’ [14] by replacing discharge with death and length of stay with age. Andersen [14] considered also a multistate model, the classical illness-death model, in order to study different statistical variants and extensions of our approach *A*_4_ including time-inhomogeneous Markov models, censoring and semi-Markov models. Approaches *A*_1_- *A*_3_ are not considered by Andersen and complement his considerations.

This study has following limitations. First, we focused on the basic approaches and did not consider any other covariates such as characteristics from the patient-, hospital- or even country-level (for instance, as in Stewardson et al. [15]). Even though there exists regression models [16] which allows for adjusting the change of length of stay (approach *A*_4_), we believe that the choice of the fundamental approach has a much stronger impact on the results than the adjustment for covariates. For instance, previous studies indicated that the time-dependent bias could not be redeemed by adjustment of several patient-level covariates [4]. Second, the hazard rates are often not time-homogeneous in real-data settings. Even though time-inhomogeneous approaches exist, we are convinced that this simplification is required to provide a clear transparency which leads to a better understanding of basic distinctions. Third, we combined the diametrically opposed endpoints discharge (alive) and death. We think that this combination is reasonable if the focus is on length of stay and their related economic costs, the topic of this paper. However, as rapid death results in shorter LOS, a length-of-stay analysis should always be accompanied with an analysis with respect to mortality. This can be done by using an extended multi-state model that distinguishes between inpatient death and discharge alive [5, 6].

## Conclusion

We conclude that a clear distinction between different estimands is needed to better understand apparently large discrepancies in the literature. We recommend the use of approaches which differentiates between pre- and post-infection time.

## Data Availability

Data are publicly available in the R-package etm (https://cran.r-project.org/web/packages/etm/index.html).

## References

[1] Lambert ML, Suetens C, Palomar M, Hiesmayr M, Morales I, Savey A (2011). Clinical outcomes of health-care-associated infections and antimicrobial resistance in patients admitted to European intensive-care units: a cohort study. Lancet Infect Dis.

[2] Barnett AG, Beyersmann J, Allignol A, Rosenthal VD, Graves N, Wolkewitz M (2011). The Time-Dependent Bias and its Effect on Extra Length of Stay due to Nosocomial Infection. Value Health.

[3] Nelson RE, Nelson SD, Khader K, Perencevich EL, Schweizer ML, Rubin MA (2015). The Magnitude of Time-Dependent Bias in the Estimation of Excess Length of Stay Attributable to Healthcare-Associated Infections. Infect Control Hosp Epidemiol.

[4] Beyersmann J, Kneib T, Schumacher M, Gastmeier P (2009). Nosocomial infection, length of stay, and time-dependent bias. Infect Control Hosp Epidemiol.

[5] Wolkewitz M, von Cube M, Schumacher M (2017). Multistate Modeling to Analyze Nosocomial Infection Data: An Introduction and Demonstration. Infect Control Hosp Epidemiol.

[6] von Cube M, Schumacher M, Wolkewitz M (2017). Basic parametric analysis for a multi-state model in hospital epidemiology. BMC Med Res Methodol.

[7] Bootsma MC. Mathematical studies of the dynamics of antibiotic resistance, PhD thesis; 2005. www.staff.science.uu.nl/boots102/PhD-thesis٪20Bootsma.pdf.

[8] Schulgen G, Schumacher M (1996). Estimation of prolongation of hospital stay attributable to nosocomial infections: new approaches based on multistate models. Lifetime Data Anal.

[9] Bernoulli D, Blower S (2004). An attempt at a new analysis of the mortality caused by smallpox and of the advantages of inoculation to prevent it. Reviews in medical virology.

[10] Allignol A, Schumacher M, Beyersmann J (2011). Estimating summary functionals in multistate models with an application to hospital infection data. Computational statistics.

[11] Allignol A, Schumacher M, Beyersmann J (2011). Empirical Transition Matrix of Multi-State Models: The etm Package. Journal of Statistical Software.

[12] Wolkewitz M, Vonberg RP, Grundmann H, Beyersmann J, Gastmeier P, Barwolff S (2008). Risk factors for the development of nosocomial pneumonia and mortality on intensive care units: application of competing risks models. Crit Care.

[13] de Vries EN, Ramrattan MA, Smorenburg SM, Gouma DJ, Boermeester MA (2008). The incidence and nature of in-hospital adverse events: a systematic review. BMJ Quality & Safety.

[14] Andersen PK (2017). Life years lost among patients with a given disease. Statistics in medicine.

[15] Stewardson AJ, Allignol A, Beyersmann J, Graves N, Schumacher M, Meyer R (2016). The health and economic burden of bloodstream infections caused by antimicrobial-susceptible and non-susceptible Enterobacteriaceae and Staphylococcus aureus in European hospitals, 2010 and 2011: a multicentre retrospective cohort study. Euro Surveill.

[16] Bluhmki T, Allignol A, Ruckly S, Timsit JF, Wolkewitz M, Beyersmann J (2018). Estimation of adjusted expected excess length-of-stay associated with ventilation-acquired pneumonia in intensive care: A multistate approach accounting for time-dependent mechanical ventilation. Biometrical Journal.

